# Landscape-scale endophytic community analyses in replicated grapevine stands reveal that dieback disease is unlikely to be caused by specific fungal communities

**DOI:** 10.1128/aem.00782-25

**Published:** 2025-06-20

**Authors:** Vinciane Monod, Valérie Hofstetter, Olivier Viret, Vivian Zufferey, Katia Gindro, Daniel Croll

**Affiliations:** 1 Laboratory of Evolutionary Genetics, Institute of Biology, University of Neuchâtel 30780, Neuchâtel, Switzerland; 2 Plant Protection, Mycology, Agroscope 419060, Nyon, Switzerland; 3 Département de l’Economie, de l’innovation et du sports (DEIS), Direction générale de l’agriculture, de la viticulture et des affaires vétérinaires (DGAV), Morges, Switzerland; 4 Viticulture, Agroscope 419060, Pully, Switzerland; Royal Botanic Gardens, Surrey, United Kingdom

**Keywords:** amplicon sequencing, fungal communities, grapevine, esca, dieback disease

## Abstract

Tree diebacks are complex and multifactorial diseases with suspected biotic and abiotic components. Microbiome effects on plant health are challenging to assess due to the complexity of fungal and bacterial communities. Grapevine wood dieback is the main threat to sustainable production worldwide, and no causality with microbial species has been established despite long-standing claims of fungal drivers. Here, we aimed to test the hypothesis that grapevine esca disease progression has reproducible drivers in the fungal species community. For this, we analyzed a set of 21 vineyards planted simultaneously with a single susceptible cultivar to provide unprecedented replication at the landscape scale. We sampled a total of 496 plants at the graft union across vineyards in 2 different years to perform deep amplicon sequencing analyses of the fungal communities inhabiting grapevine trunks. The communities were highly diverse with a total of 4,129 amplified sequence variants assigned to 697 distinct species. We detected trunk fungal community shifts over years of sampling, vineyards and climatic conditions, as well as disease status. However, we detect no specific fungal species driving symptom development across vineyards, contrary to long-standing expectations. The high degree of environmental standardization in the decade-long experimental plots and the well-powered replication provide the clearest evidence yet that grapevine wood dieback is most likely caused by environmental factors rather than specific pathogens. Furthermore, our study shows how landscape-scale replicated field surveys allow for powerful hypothesis testing for complex dieback disease drivers and prioritize future research directions.

**IMPORTANCE:**

Tree diebacks are complex diseases suspected to be caused by both biological and environmental drivers. Grapevine wood dieback is a major threat to vineyards worldwide, but no specific microbial species have been experimentally implicated, despite claims that fungi are causing the symptoms. Here, we tested whether the progression of grapevine esca disease is driven by specific fungal species. We analyzed 21 long-established vineyards planted at the same time with the same susceptible grape variety to ensure consistent conditions. Over the years, we observed changes in the fungal communities inhabiting the trunk depending on the vineyard, climate, and disease status. However, contrary to expectations, we did not detect any specific fungal species that consistently could cause symptoms across the vineyards. The high level of environmental control and replication in our study provides strong evidence that grapevine wood dieback is more likely caused by environmental factors rather than specific pathogens.

## INTRODUCTION

Dieback is the deterioration of tree health observed increasingly in forests and perennial crops constituting a global concern (1–3). Environmental warming is a key factor in increasing the dieback likelihood and favors the spread of plant diseases (4, 5). Tree diebacks are complex and multifactorial diseases with biotic and abiotic components (6, 7). Determining the combination of factors leading to decline is challenging (8). Vascular wilts are among the most destructive tree declines (9). Complex biotic interactions, including polymicrobial and insect activity, influence the onset of dieback and increase severity (1). Even though most plant diseases are thought to be caused by discrete pathogen species, there is growing evidence that complex plant diseases can arise from synergistic interactions among multiple microorganisms (1, 10). Given the complexity of the microbial communities associated with perennial plants, investigating links between microbiome composition and disease status is essential.

Microbiome assembly effects on perennial plant health and dieback rates are challenging to assess due to the large number of coexisting microbes including endophytic fungi (11). Members of fungal communities interact with each other and with their hosts to cause a wide range of beneficial or pathogenic effects (12). The microbiota, by providing additional ecological functions to the host (13), plays a crucial role in plant adaptation to biotic and abiotic environmental conditions, potentially enhancing plant health and stress resistance (14, 15). Microbial communities can promote plant growth by simulating water and nutrient intake and increase health through antibiosis against pathogens and pests (14, 16–19). The spectrum of symbiotic associations and their consequences is not well-defined and depends on environmental conditions. These associations can transition between commensalism, mutualism, or parasitism (20). Environmental factors and host genotype may also influence the lifestyle of fungi transitioning from the endophyte to pathogen (21). Some endophytes display a latent state and turn symptomatic when the plant encounters stress conditions such as drought, humidity, or nutrient starvation (20). Fungal endophytes comprise a diverse group of species, some of which are known to cause plant diseases as pathogens, while also being present on asymptomatic plants (18, 22). The mechanisms by which endophytes transition from commensalism or mutualistic interactions to becoming pathogens remain poorly understood (23, 24).

The host microbiome can undergo substantial changes in community structure in the presence of pathogenic species and disease progression (24, 25). For instance, in olive orchards suffering from anthracnose, lower endophyte diversity is observed with higher disease incidence (11). Synergism among different pathogens can increase disease severity in various tree species including apple, chestnut, hazelnut, and grapevine (10). The impact on tree health resulting from microbial interactions with sequential or cumulative effects can also be modulated by abiotic factors. Decline diseases, where both abiotic and biotic interactions contribute significantly to disease development, need to be addressed with an integrated system approach (1).

Grapevine wood dieback is considered the main threat to sustainable grapevine cultivation worldwide but is caused by yet unresolved factors (26). A range of wood-colonizing fungal pathogens was suggested to contribute to disease progression (27–30), in addition to changes in climatic and soil conditions (31–34). The main form of dieback is identified as grapevine trunk disease (GTD) with significant impacts on yield and reduced fruit quality, leading to high plant replacement rates and economic losses (30, 35). GTD is classified into several disease types (35) including the most damaging esca (36). Esca includes trunk necrosis development in mature vines, along with foliar symptoms and/or symptoms on the shoots with grape wilting. The expression of foliar symptoms can be discontinuous, but plants usually die within a few years following the onset of initial symptoms (37, 38). The discontinuous expression of the disease suggests complex interactions with potential pathogenic species and environmental conditions (39). Esca disease is thought to be associated with the activity of three distantly related fungi (40): *Phaeoacremonium* spp., *Phaeomoniella chlamydospora*, and *Fomitiporia mediterranea*, considered the most serious pathogens of vines and are the main agents of vascular disease in Europe (30, 39, 41). Members of the Botryosphaeriaceae family are also considered to play a role in the disease complex (30, 35). These fungi have consistently been isolated from symptomatic grapevines, displaying a close association with esca symptoms such as foliar necrosis and wood discoloration (28, 42). However, fungal species isolated from symptomatic plants often occur both on symptomatic and asymptomatic plants, suggesting that the disease is not solely triggered by the presence of specific species (43–45). Shared occurrence of fungal species in both symptomatic and asymptomatic plants suggests a potential endophytic phase (35, 46). The exact mechanisms and interactions between these fungi and the grapevine host remain poorly understood (30). Whether the association of fungal species with symptom development of esca is based on causal relationships remains unknown (30, 47). The major limitation of the system is that disease symptoms cannot be reproduced in controlled infections (48).

Here, we aimed to test the hypothesis that grapevine esca disease progression is trackable by reproducible driver species among fungal communities inhabiting trunks. High-throughput amplicon sequencing techniques can generate high-resolution assessments of fungal diversity within grapevine trunks (14, 49, 50). Examining changes in microbiome composition as a function of symptom development in various environments helps pinpoint species potentially involved in the disease. To achieve this, we analyzed a replicated set of 21 vineyards, all planted simultaneously with a single susceptible cultivar (i.e., Gamaret) at the landscape scale. In a previous study, we estimated that esca disease is present in the plot network with varying incidence from one season to the next and across different vineyard locations. We observed that the abiotic soil type and early summer rainfall conditions correlated with a higher incidence of esca (33). In this study, we aim to investigate the fungal load and its potential link to the disease’s presence at this landscape scale. To achieve this, we collected samples from both asymptomatic and symptomatic plants in each vineyard across two different years to conduct amplicon sequencing of the grapevine trunk fungal communities. Sampling was performed at the trunk level as fungi associated with esca disease are usually only detected in the trunk (51). We analyzed mycobiome composition partition within and across vineyards to assess community stability in the absence of disease. Using repeated assessment of asymptomatic and symptomatic plants detected in vineyards, we aimed to investigate potential associations of specific taxa with disease symptom expression or mycobiome association with geographical locations that display various degrees of esca incidence.

## RESULTS

### Replicated assessment of the trunk mycobiome across vineyards

**Fig 1:**
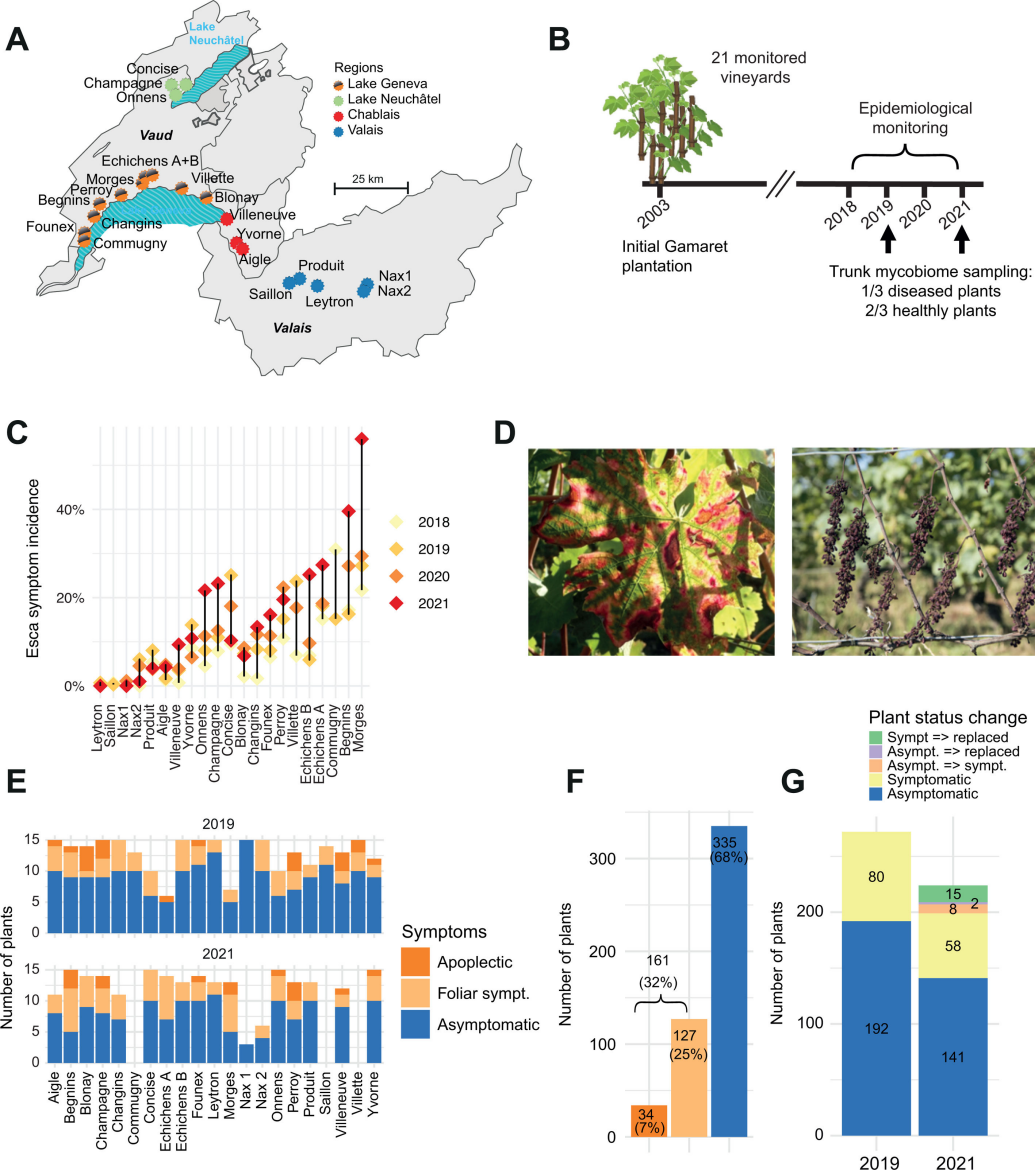
Collection of vine trunk samples to survey the mycobiome community composition. (**A**) Location of the studied vineyards (*n* = 21) in Western Switzerland, colored according to the main viticultural regions. (**B**) Life history of the studied vineyards with planting in 2003, followed by the monitoring of esca-BD symptoms (2018–2021) and the sampling seasons (2019 and 2021). (**C**). Annual incidence of esca foliar symptoms in the studied vineyards. (D) Esca symptoms: typical foliar symptoms ("tiger-stripes") and apoplectic symptoms with wilting of the whole plant (adapted from reference 33). (**E**) Number of samples successfully sequenced according to categories. (**F**) Proportion of plants sampled by category (asymptomatic plants *n* = 335, symptomatic plants with leaf symptoms *n* = 127, and symptomatic plants with apoplexy *n* = 34). (**G**) Overview of sequenced plants by symptom category and sampling year. The temporal sequence of the health status refers to the observed status in 2019 and 2021.

We analyzed 21 vineyard plots planted simultaneously in 2003 with *Vitis vinifera* cv. Gamaret in Western Switzerland (Fig. 1A). All plants originate from a single nursery to ensure standardization of both age and genetic makeup. The set of replicated Gamaret plots was tracked based on physiological indicators such as yield, must and leaf chemical composition, and plant water status, along with meteorological and climatic recordings, soil analyses, and the incidence of esca (52). Over a span of 4 years (2018–2021) and at the end of summer when symptoms are most visible, we recorded esca presence plant-by-plant on each site. When a plant showed esca symptoms for several years and then was later replaced, we assumed that this was due to the progression of esca disease. Esca foliar incidence varied between sites and years, ranging from 0% (no affected plants) to over 50% in some locations (Fig. 1C). To determine whether the grapevine trunk fungal community could explain the prevalence of esca, we sampled vine plants in the plot network in 2019 and 2021 (Fig. 1B). We randomly selected 10 asymptomatic and five symptomatic plants showing either foliar symptoms or apoplexy at the end of the season when symptoms are clearly visible (Fig. 1B and D). Where possible, we sampled the same plants for the 2 years (unless the plant had died in the meantime). We used an optimized protocol (43) to obtain wood cores at the grafting point for each plant (49). To barcode the endophytic fungal community present in the wood cores, we amplified the ITS with primer pairs ITS1F-ITS4. Previous work on the mycobiome of grapevine trunks showed that utilizing the ITS fragments alone offered a better trade-off between the depth of coverage and taxonomic resolution compared to analyzing a longer fragment, including also segments of the 28S ribosomal gene sequence (49).

We successfully amplified 496 samples over all sites (Table S1). Samples with low PCR yield were excluded, as well as samples from sites where the vineyard was uprooted during the sampling period (see Materials and Methods for details). We generated PacBio circular consensus sequencing (CCS) data for 192 asymptomatic and 80 symptomatic plants for the 2019 sampling period (Fig. 1G). In 2021, 141 asymptomatic plants and 58 symptomatic plants were sampled. For the plants without replacement between 2019 and 2021, 141 remained asymptomatic and 58 symptomatic. 30 asymptomatic plants were not sampled again in 2021 due to the exclusion of vineyards with highly variable management practices. Additionally, we observed that eight plants initially asymptomatic in 2019 became symptomatic (Fig. 1G). Furthermore, the plot owners uprooted two asymptomatic and 15 symptomatic plants from 2019. In 2021, we randomly selected new plants as replacements. The composition of the sampled plants, including proportions of asymptomatic and symptomatic individuals, varies among plots, in particular for esca symptom categories (Fig. 1E). Overall, we sequenced 335 asymptomatic plants (68%) and 161 symptomatic plants (32%) (Fig. 1F).

**Fig 2:**
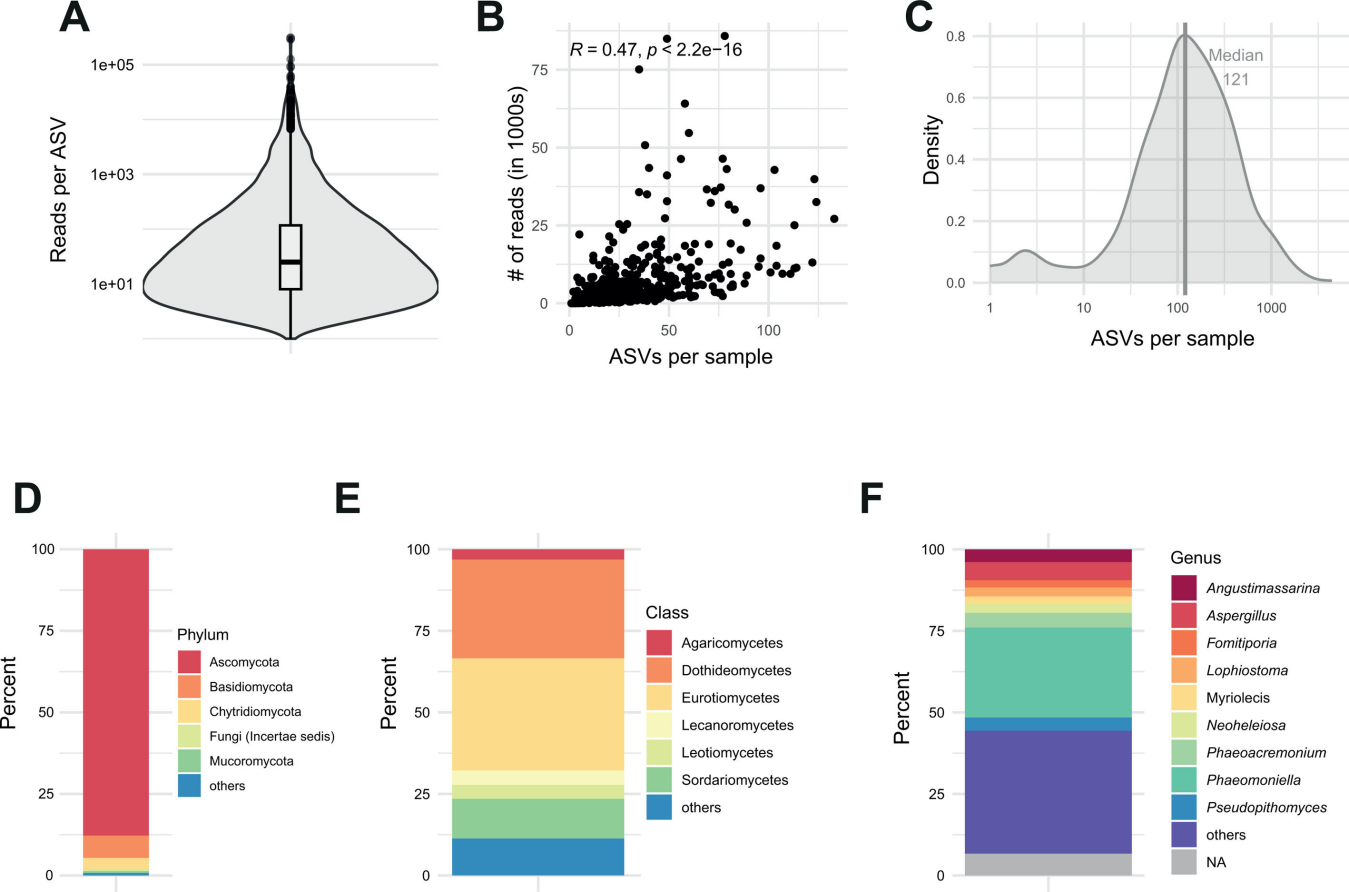
Amplified sequence variants (ASV) recovery and diversity. (**A**) Recovered reads per ASV (median = 25 reads). (**B**) Relationship between the number of raw reads and the ASVs detected per sample. (**C**) Distribution of ASVs per sample (median = 121 ASVs). (**D**) Proportion of phyla represented by the amplicon sequences (“others”: phyla with <0.5%). (**E**) Proportion of classes in the total sequences ("others": classes with <2%). (**F**) Proportion of genus in the total sequences (“others”: comprises classes with <2% frequency).

We analyzed 3,390,060 CCS reads after quality filtering steps with a mean of 6,834 reads per sample. We detected no meaningful difference in per-sample read frequencies according to the asymptomatic and symptomatic status (*t*-test; *P* > 0.05). The reads clustered into 4,129 distinct amplicon sequence variants (ASVs), assigned to 697 species based on matches in the UNITE fungal ribosomal DNA database (53). The median number of reads per ASV was 25 (Fig. 2A). Most ASVs were rare, with 30% of the ASVs having 10 reads or less. We obtained a median of 121 ASVs per sample (Fig. 2C). The number of detected ASVs per sample was correlated with the number of reads (*r* = 0.47, *P* < 0.05; Fig. 2B; Table S2). Overall, the recovered endophytic community was composed of Ascomycota (87.8%), Basidiomycota (*6.82%),* Chytridiomycota (3.29%), and others (2.1%). Among the 41 identified classes, the most abundant classes were Eurotiomycetes (34.4%), Dothideomycetes (30.3%), Sordariomycetes (12.2%), Lecanoromycetes (4.45%), and Leotiomycetes (4.21%). Among the 497 detected genera, we found a dominance of *Phaeomoniella* (27.6%), *Aspergillus* (5.55%), *Phaeoacremonium* (4.50%), *Pseudopithomyces* (4.01%), and *Angustimassarina* (3.90%) (Fig. 2D, E and F; Table S3).

### Fungal microbiome structure among healthy and symptomatic plants

**Fig 3:**
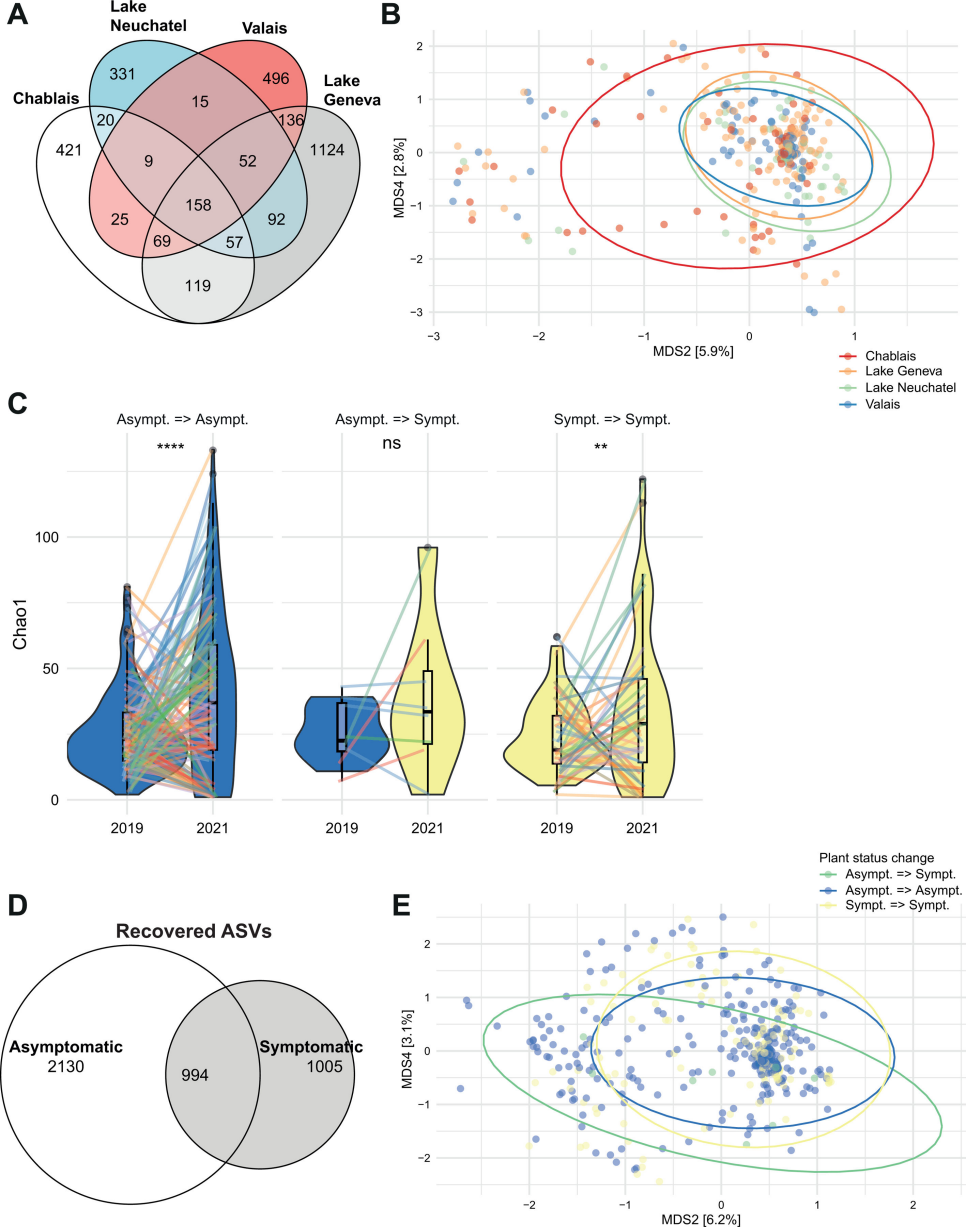
Diversity of asymptomatic or symptomatic plant mycobiomes. (**A**) Proportion of ASVs shared among asymptomatic plants per geographic regions. (**B**) Principal coordinate analysis (PCoA, no transformation, Bray-Curtis distance on ASV diversity, *n* = 3,124) of mycobiome diversity of asymptomatic plants across geographic regions. Each point represents the mycobiome composition at the ASV level of the trunk of once-sampled vine plants. (**C**) Violin plots displaying the α-diversity on esca asymptomatic and symptomatic sampled plants (Chao1 index) taking into account their epidemiological history (Asympt => Asympt: plants that remained asymptomatic during the 2 years of sampling; Asympt => Sympt: plants that changed from asymptomatic in 2019 to symptomatic in 2021; Sympt_Sympt: plants recorded as symptomatic during the 2 years of sampling) with individual samples linked and color-marked by vineyard. (**D**) Proportion of ASVs shared between asymptomatic and symptomatic sampled plants. (**E**) Principal coordinate analysis (PCoA, no transformation, Bray-Curtis distance on ASV diversity, *n* = 4,169) of the mycobiome diversity of the samples.

Plants are typically associated with diverse microbiomes independent of their health status. To assess the fungal microbiome structure of asymptomatic plants, we analyzed the 502 fungal species detected in asymptomatic grapevine trunks. The diversity of the recovered mycobiome varied between the two sampling years with 1–133 ASVs recovered per plant and a total of 3,124 ASVs. The total diversity between the two sampling years was comparable, with 351 species recovered in 2019 (1,751 ASVs; *n* = 192 samples) and 374 species in 2021 (2,057 ASVs; *n* = 143 samples. The mycobiome was only weakly shared between regions across Western Switzerland, with 158 (3.8%) out of 4,129 ASVs found in all regions (Fig. 3A). If we consider the proportions of reads associated with each ASV, fungal communities are more similar, with 64% of reads assigned to the same taxa across geographical regions. Differences in mycobiome composition between regions were mostly due to rare ASVs. *P. chlamydospora* was the most abundant species in all regions. A principal coordinate analysis (PCoA) of the mycobiome revealed substantial overlaps among regions and vineyards, yet fungal communities differ significantly among the regions (PERMANOVA, *R*^2^ = 0.016, *P* = 0.001) and among vineyards (PERMANOVA, R2 = 0.096, *P* = 0.001). The PCoA highlights the substantial mycobiome variability among plants, vineyards, and regions and the challenge to test for consistent species occurrences across fungal communities (Fig. 3B).

Symptomatic plants (foliar and apoplexy symptoms) did not differ significantly from asymptomatic plants in recovered species or ASV diversity with 502 species (3,124 ASVs) detected among asymptomatic plants (*n* = 335) and 418 species (1,999 ASVs) detected among symptomatic plants (*n* = 161) (ANOVA, *P* > 0.05). Comparisons of Chao1 diversity among different sampling years and various plant health status categories revealed significant differences between plants remaining healthy (i.e., asymptomatic) or keep showing symptoms between the sampling years (Wilcoxon *P* = 1.6e^−5^ for asymptomatic plants; *P* = 0.061 for symptomatic plants; Fig. 3C). Variability in the recovered diversity is not associated with the health status of the sampled plant. The fungal diversity recovered for the same plant varied across the two time points, but differences in diversity for plants turning from asymptomatic to symptomatic across sampling years were not significant (Chao1 diversity index; Wilcoxon *P* > 0.5; Fig. 3C). However, this assessment is based on a comparatively low number of observations (*n* = 8). Asymptomatic and symptomatic plants shared overall 24% of ASVs (Fig. 3D). If we consider the proportions of reads associated with each ASVs, fungal communities are more similar, with 89% of reads assigned to the same taxa between health status, even if the sample size of asymptomatic and symptomatic plants differs with more asymptomatic plants sampled. Differences in fungal community composition across samples of different health status were largely due to rare taxa. The PCoA revealed no obvious clustering between plants remaining asymptomatic, persistent in a symptomatic stage, or turning symptomatic over the sampling period (Fig. 3E). Community composition was nevertheless significantly different between symptomatic and asymptomatic plants (PERMANOVA, *R*^2^ = 0.003, *P* = 0.014). Fungal community composition was significantly different between asymptomatic plants compared to plants suffering from apoplexy (PERMANOVA, *R^2^* = 0.00465, *P* = 0.005). Fungal communities of plants exhibiting either foliar symptoms or apoplexy were not significantly different (PERMANOVA, *P* > 0.05). However, we must remain cautious as this may be influenced by unequal sample sizes as the total diversity recovered will be greater for asymptomatic plants for which we have more replicates.

### Taxonomic profiles highlight taxa linked to asymptomatic plants

**Fig 4:**
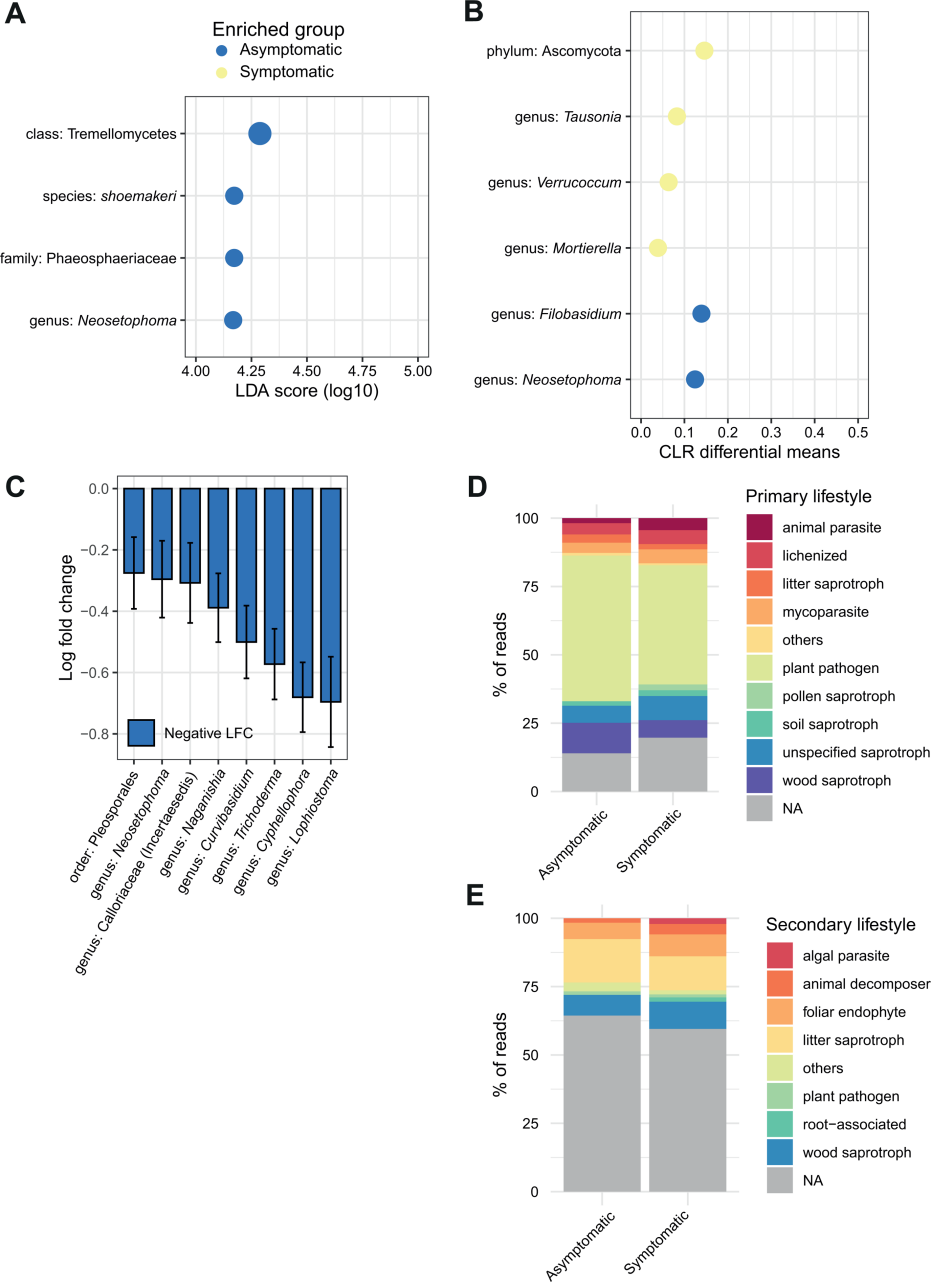
Identification of differentially abundant taxa in the mycobiome of asymptomatic and symptomatic plants. (**A**) Linear discriminant analysis (LEfSe) was used to identify overabundant taxa in asymptomatic and symptomatic plants (CLR normalization, *P*-value < 0.05). Enriched taxa in the asymptomatic group are shown in blue and enriched taxa in the symptomatic group in yellow. The list of discriminating features according to the classes (asymptomatic and symptomatic) is ordered by the magnitude of the effect with which they differentiate the classes. (**B**) Microbiome composition analysis (ANCOM) identified compositional differences (*P*-value < 0.05) in the mycobiome communities of asymptomatic and symptomatic sampled plants. Enriched taxa in the asymptomatic group are shown in blue and enriched taxa in the symptomatic group in yellow. (**C**) Analysis of microbiome composition represented by effect size (log fold change) and 95% confidence interval bars (two-sided; Bonferroni adjusted) derived from the ANCOM-BC model. (**D**) Trait-based approach with the proportion of primary lifestyle profiles between asymptomatic and symptomatic sampled plants. (**E**) Trait-based approach with the proportion of secondary lifestyle profiles between asymptomatic and symptomatic sampled plants.

**Fig 5:**
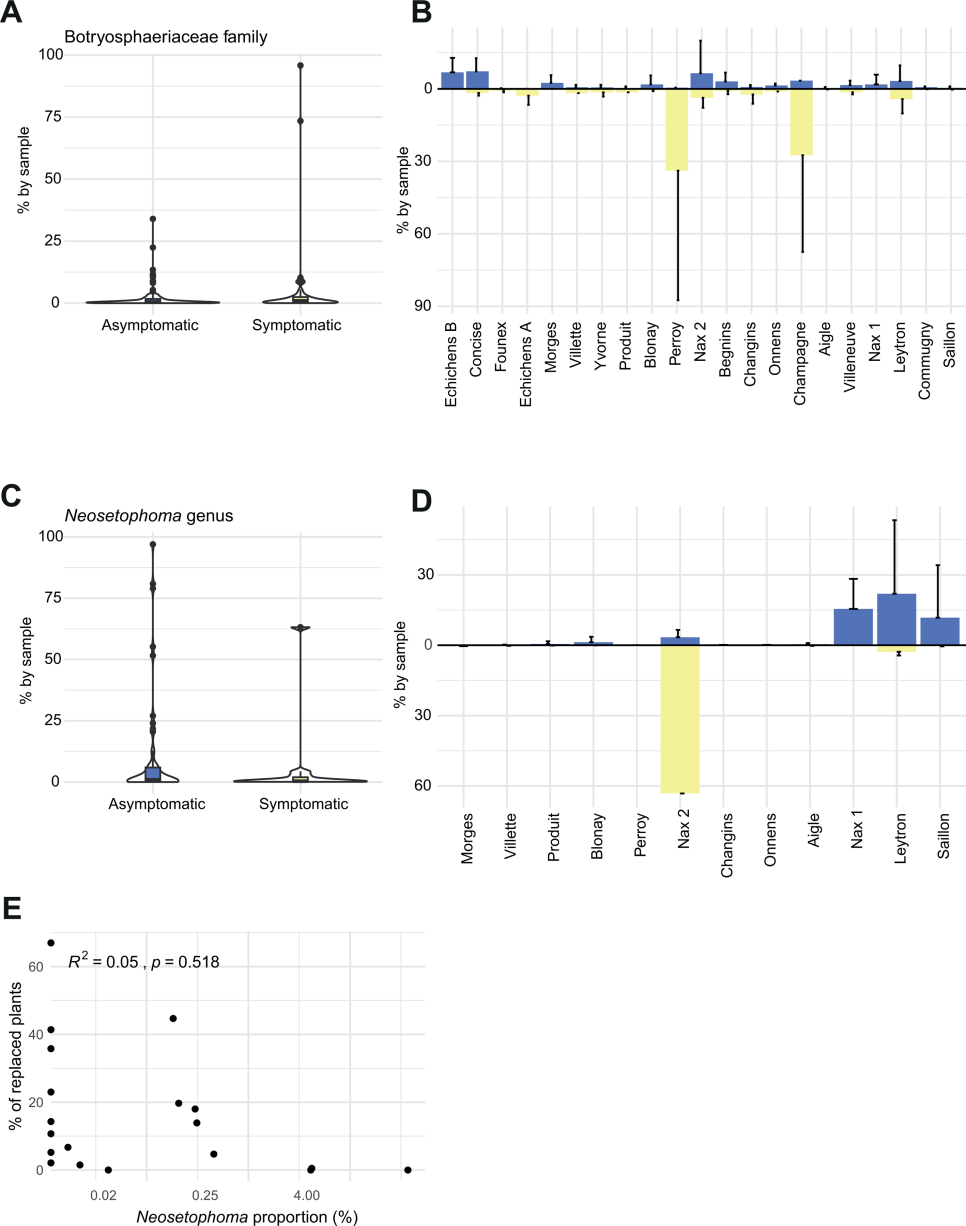
(A) Botryosphaeriaceae family with symptomatic and asymptomatic taxa. (**B**) The mean (standard deviation in black) of the Botryosphaeriaceae family proportion by vineyard for asymptomatic (blue) and symptomatic (yellow) sampled plants. (**C**) Proportion of *Neosetophoma* genus between asymptomatic and symptomatic plants. (**D**) The proportion of the *Neosetophoma* genus varies between vineyards in asymptomatic (blue) and symptomatic (yellow) sampled plants. (**E**) Relationship between the proportion of *Neosetophoma* genus by plot and the proportion of replaced plants.

We used discriminant analyses (DA) to identify significantly overrepresented taxa in both asymptomatic and symptomatic plants, aiming to pinpoint specific species underpinning the observed differences in community composition (Table S4). DA indices were constructed for a total of 496 plant samples. We used three different approaches to assess the evidence for taxa enrichment in symptomatic versus asymptomatic plant trunk mycobiomes. First, linear discriminant analysis effect size (LEfSe) analyses identified the *Neosetophoma* genus, a species of the same genus (*N. shoemaker*) and the related Phaeosphaeriaceae family and the class of Tremellomycetes as enriched in asymptomatic plants (Fig. 4A). Second, an analysis of the composition of microbiomes (ANCOM) identified six enriched genera, including two enriched in asymptomatic plants (*Neosetophoma* and *Filobasidium*) and three enriched in symptomatic plants (*Tausonia*, *Verrucoccum,* and *Mortierella*). ANCOM also identified the Ascomycota phylum as enriched in symptomatic samples (Fig. 4B). ANCOM-BC identified seven genera (*Neosetophoma*, *Calloriaceae*, *Naganishia*, *Curvibasidium*, *Trichoderma*, *Cyphellophora,* and *Lophiostoma*) and an order (Pleosporales) as having reduced abundance (negative LFC) in symptomatic compared to asymptomatic plants (Fig. 4C). Hence, the ANCOM method was the only one to identify enriched taxa in symptomatic plants. The *Neosetophoma* genus was supported by evidence and consensus between methods for association with asymptomatic plants. Proportionally, the *Neosetophoma* genus represents 0.5% of the reads of asymptomatic plants compared to 0.03% in symptomatic plants (Fig. 5C). Upon examining the distribution of the *Neosetophoma* genus across various geographical regions, a notably higher occurrence was observed in the Valais region (Fig. 5D). Valais is the region that exhibits the lowest incidence of esca impact (52). When we examined the presence of the *Neosetophoma* genus alongside the recorded mortality rates in each vineyard, we did not detect any correlation, though (R = 0.05 *P* = 0.518) (Fig. 5E).

To determine potential differences in the ecological roles of fungal communities in symptomatic and asymptomatic plant samples, we classified each identified genus into functional groups using the FungalTrait database (54). After considering predicted guilds and trophic modes, our analysis revealed no significant differentiation between asymptomatic and symptomatic plants (Fig. 4D and E). It should be noted that many genera are classified as pathogenic. This is potentially a bias of the database as pathogenic genera are more studied and described than others.

### Key genera previously associated with esca

**Fig 6:**
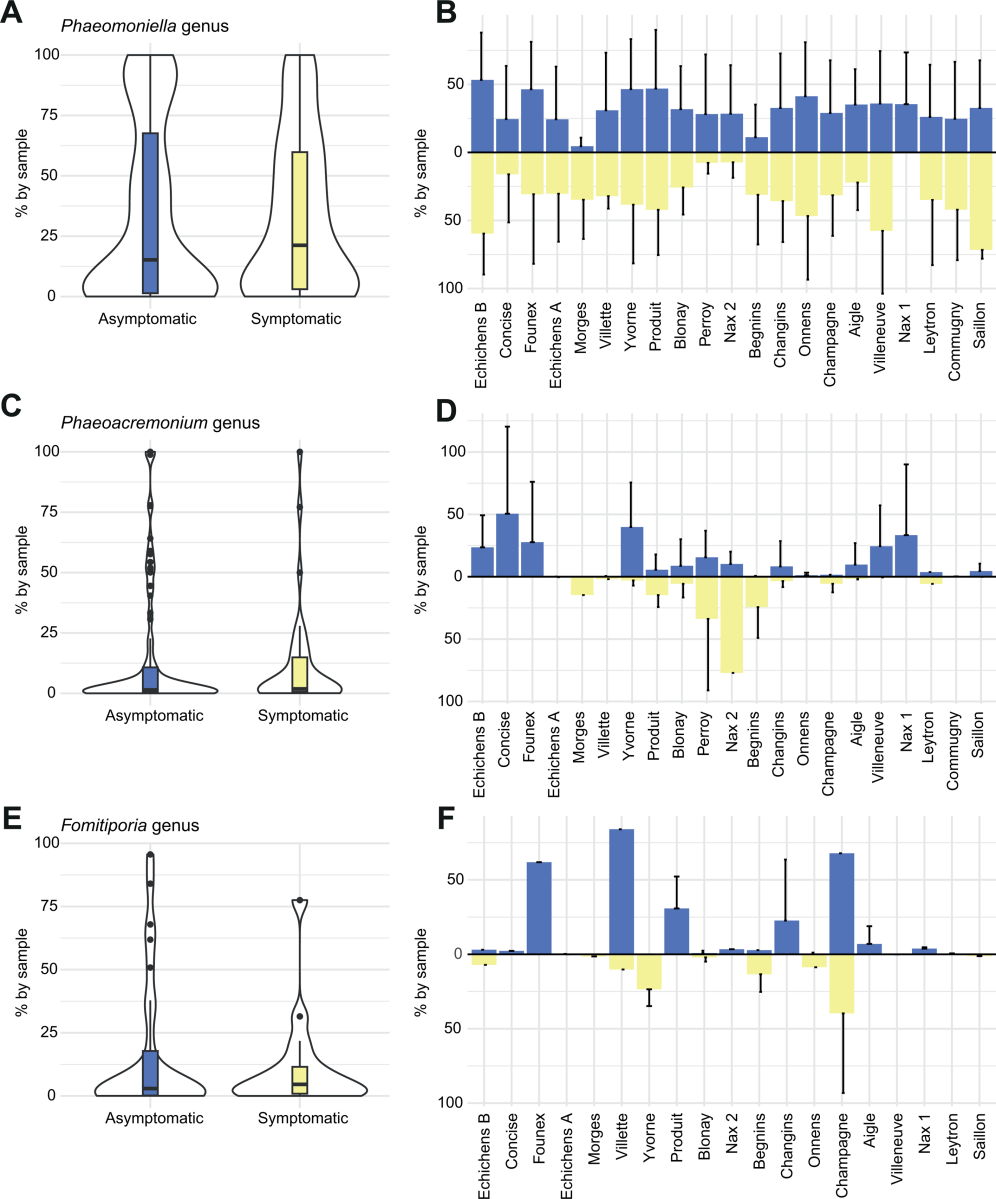
Fungal taxa commonly associated with esca per genus. (**A**) *Phaeomoniella* genus with symptomatic and asymptomatic taxa. (**B**) The mean (standard deviation in black) of the *Phaeomoniella* genus proportion by vineyard for asymptomatic (blue) and symptomatic (yellow) sampled plants. (**C**) Proportion of *Phaeoacremonium* genus between asymptomatic and symptomatic plants. (**D**) The proportion of the *Phaeoacremonium* genus varies between vineyards in asymptomatic (blue) and symptomatic (yellow) sampled plants. (**E**) Proportion of the *Fomitiporia* genus between asymptomatic and symptomatic sampled plants. (**F**) The proportion of the *Fomitiporia* genus across vineyards and asymptomatic (blue) and symptomatic (yellow) sampled plants.

We retrieved a set of taxa commonly described to be associated with grapevine trunk diseases (30, 39, 41). We focused on the presence and relative abundance of the genera *Phaeomoniella*, *Phaeoacremonium,* and *Fomitiporia*, as well as the Botryosphaeriaceae family (Fig. 5A through B; Fig. 6). We retrieved ASVs assigned to each taxonomic unit across vineyards and plant health status. We found no evidence that these taxonomic units were enriched in symptomatic plants (Fig. 6). The proportions of *Phaeomoniella* (mean of 34.1% in asymptomatic; 33.1% in symptomatic plants), *Phaeoacremonium* (mean of 14.7% in asymptomatic; 11.7% in symptomatic plants), and *Fomitiporia* (mean of 16.5% in asymptomatic; 11.6% in symptomatic plants) in both symptomatic and asymptomatic plants were comparable (Fig. 6A, C and E). Relative abundance of the focal taxa varied across vineyards, ranging for *Phaeomoniella* from 100% to 0.02% in asymptomatic and symptomatic plants, for *Phaeoacremonium* from 100% to 0.02% in asymptomatic plants and from 100% to 0.06% in symptomatic plants, and for *Fomitiporia* from 95.6% to 0.03% in asymptomatic plants and 77.5% to 0.02% in symptomatic plants (Fig. 6B, D and F).

## DISCUSSION

Woody plant decline is likely caused by a multitude of fungal species and is facilitated by environmental conditions. Here, our objective was to examine compositional differences in trunk-inhabiting fungal communities in vineyards affected to varying degrees by esca. The analysis of the vine trunk mycobiome revealed a remarkably diverse fungal community with weak differentiation at the vineyard or regional level. We found overrepresentation of several taxa in asymptomatic plants; however, no taxa were overrepresented in symptomatic plants. Additionally, key taxa typically implicated in esca did not show any significant association with plant health status.

### Extensive mycobiome variability across sampling scales

Fungal diversity among sampled plants exhibited high heterogeneity, confirming analyses conducted across various wild or cultivated plant species (55, 56). The grapevine trunk mycobiome primarily consisted of rare taxa, consistent with many host-associated mycobiome studies (44, 57–61). Variation in sample diversity may be attributed to factors such as sampling bias, disparities between plant tissues containing both living and deceased material, intra-vineyard diversity, or differences in pedoclimatic conditions (14, 56, 62). We observed dissimilarities in fungal composition across geographic areas, consistent with findings from previous studies on grapevine microbiome composition (63) and other woody plants (64). These observations suggest that fungal endophytes colonize the tissues of the hosts through a potential horizontal transfer of diversity from the surrounding environment via soil- or airborne spores (61, 65, 66).

Alpha diversity can decline with increasing disease symptom severity, suggesting that reduced diversity, as shown in other systems, may play a protective role mediated by the remaining species (67). Similar findings were obtained from acute oak decline (68), from fungal root pathogens (69), or bumble bees (70). The opposite was also observed, though, in pine wilt disease (64) or ash dieback (71), where a higher diversity of the microbiome was observed along with symptom severity. Higher microbiome diversity is thought to stem from the pathogen suppressing plant resistance mechanisms and, thereby, facilitating the colonization by other microorganisms (64). Plants affected by esca showed neither a decrease nor an increase in alpha diversity in our study. No significant differences in alpha diversity of declined and healthy trees were found in key tree species (holm oak, cork oak, chestnut, and Pyrenean oak) of the Mediterranean forest (72). While richness in diversity remained unchanged, alterations in community composition could still have occurred depending on the health status of the plant. However, the interpretation of the functional role of the fungal community can be challenging because fungal species can undergo lifestyle transitions depending on the environment (22, 73). In addition, the esca symptom development is nonlinear, with plants displaying symptoms inconsistently from season to season. This variability makes it challenging to relate health status to the composition of the fungal community. The inconsistency in esca symptoms within a single plant suggests that the fungal community can impact the plant’s health even when surface symptoms are not apparent. Furthermore, considering that the fungal species associated with esca disease are typically detected in the trunk, far from the leaf symptoms, this creates further challenges to correlate the presence or absence of specific species with visible symptoms. We also caution that the absence of detected diversity shifts between asymptomatic and symptomatic plants could have been affected by the lower number of available symptomatic plants.

### Grapevine fungal community structures are shaped by rare taxa

Despite no overall diversity effects, we detected a broad range of variations in the community composition between asymptomatic and symptomatic plants. However, plant health was not a strong enough factor to reveal distinct community effects using beta dissimilarity analyses. The imbalance of sample size between symptomatic and asymptomatic plants may have affected the power of detection for shifts in community composition. Across geography and esca health status, we observed high intersample variability. Such variability in the host-associated mycobiome creates significant challenges to pinpoint cryptic species underpinning diseases. Nonetheless, many environmental microbiome communities are typically characterized by the presence of a long tail of rare taxa (60, 74), and the mechanisms through which environmental conditions shape the pathobiome remain largely unexplored (75). Overcoming statistical limitations in associating rare taxa with disease development would require either substantially expanding the sampling effort or reducing environmental noise.

### No differentiated fungal community associated with symptomatic plants

We conducted differential abundance analyses to assess the enrichment of particular taxonomic groups in symptomatic plants using three distinct approaches but found no strongly associated taxa. Nevertheless, our power of detection would have been maximized under evenness of the sample size between asymptomatic and symptomatic plants. This is in line with results of previous research on fungal trunk communities affected by esca, revealing no direct association between specific taxa and symptomatic esca plants (43, 44, 76). We found no distinct microbiome associations with the different vineyard regions, despite observing a striking gradient of esca severity across these areas. Our study builds upon previous research by substantially increasing the number of samples, emphasizing that even sampling strategies with hundreds of data points may still be underpowered. Previous research conducted on the same vineyards has linked the incidence of esca symptoms to pedo-climatic factors (52), suggesting that soil water-holding capacity is a key factor for disease development. The soil retention capacity is influenced by the amount of precipitation and various soil characteristics. Whether such soil properties are causal or merely show correlated responses to an, as yet, unknown factor remains uncertain. If soil properties are indeed the root cause of the disease, a number of fungal taxa may in turn sporadically associate with particular soil types without playing a relevant role in the disease. Furthermore, any association of endophyte taxa may similarly be due to correlations between soil characteristics and fungal diversity (77). Furthermore, endophytes may transition from a latent asymptomatic state to an active state after the plant encountered stress conditions such as drought, humidity, or nutrient starvation (20). This transition presents a promising field for future research, although the remaining challenges are substantial. An interesting observation was the significant enrichment of the *Neosetophoma* genus in asymptomatic plants, as supported by a consensus among all differential abundance methods. This genus is most prevalent in the Valais region, which also exhibits the lowest incidence of esca (33). However, it is worth noting that the *Neosetophoma* genus is absent from numerous analyzed vineyards. Therefore, the strong relationship between the presence of the *Neosetophoma* genus and the absence of esca symptoms should be interpreted cautiously. Rather than acting as a potential protective agent, *Neosetophoma* might merely be a species prevalent in regions less affected by esca. However, it is conceivable that endophytes residing in woody plants play a defensive role for the host plant by producing a range of protective mycotoxins and enzymes (14, 15). Lack of associated taxa with trunk disease does not negate causal interactions of fungal taxa with the disease but rather suggests that the complexity of biotic drivers is too high. In addition, the interplay between biotic factors and abiotic conditions, such as environmental stressors, warrants further investigation to fully understand their combined impact on trunk disease dynamics. We also focused on taxa traditionally associated with esca, specifically the genera *Phaeomoniella*, *Phaeoacremonium*, and *Fomitiporia*, as well as the Botryosphaeriaceae family. We detected no differential abundance for these taxa, regardless of whether the plants were asymptomatic or symptomatic. Since these fungi were found only in the trunk and not near leaves, where the symptoms were observed, this suggests that either these fungi are not involved in esca pathogenesis or that the production of toxins by these fungi, creating the foliar symptoms, is triggered by certain abiotic conditions.

Plant health or disease should not be viewed as a binary concept but rather an expression of symptoms along a continuum. In complex diseases like tree dieback, multiple factors are likely involved, and resolving causal relationships between taxa and health is challenging. The presence of fungal endophytes residing within plants without causing harm challenges our traditional understanding of plant infection processes and how causal taxa should be identified (20). The ecological relevance of rare species is increasingly recognized with key functions in host-associated microbiomes (78). Yet, determining perturbations caused by rare species is challenging as most studies were likely underpowered (79). Further research under more controlled conditions is needed to determine what disruption or imbalance in the plant microbiome is considered detrimental to plant health (80, 81) and what meaningful boundaries can be drawn between endophytes and pathogens (82).

## MATERIALS AND METHODS

### Sample collection

Wood samples (*n* = 585) were collected in August 2019 and 2021 from vineyards located in four viticultural regions in western Switzerland (Fig. 1A and B). A total of 21 vineyards planted with a single grapevine variety (Gamaret) were sampled. Gamaret originates from a cross between Gamay and Reichensteiner varieties (*V. riparia* X *V. rupestris*) grafted onto 3309C rootstock. All plants originate from the same nursery (Les frères Dutruy SA; Founex, Switzerland) and were planted in 2003 as a part of a study to evaluate the plasticity of grapevine varieties across the viticultural region of western Switzerland (83). Winegrowers followed homogeneous management and cultural practices to avoid bias in the results. The 21 grapevine plots were maintained under similar viticultural management based on the Guyot training system. These plots represent repetitions, arranged across several pedological units. Beyond the aspect of plant homogeneity, these vineyards were selected due to their variable rate of esca expression (33). Plant mortality was assumed if esca symptoms were observed prior to replacement. Since 2018, a whole plant-by-plant monitoring of esca symptoms has been carried on all the 21 plots. We recorded typical foliar esca symptoms with a tiger-stripe pattern and the acute form characterized by the wilting of the entire plant called apoplexy. Our survey of the vines was carried out at the end of the summer, when the symptoms are fully developed, reducing the risk of confusion with another disease. In the monitoring, we differentiate foliar symptoms from apoplectic form. However, we only considered the plant as being asymptomatic or symptomatic regarding the mycobiome analyses. Asymptomatic plants did not show any signs of symptom expression prior to and during the study. In addition, for replacements made before 2018, growers also recorded the appearance of symptoms and the likelihood of esca involvement. These data enable us to determine the mortality rate due to esca within each vineyard. In each vineyard, five symptomatic plants displaying the typical foliar esca symptoms, including leaf discoloration, tiger-stripe pattern, or plant wilting (28), were collected alongside ten asymptomatic plants (no observed symptoms since 2018) in 2019 and 2021. Plants were 16 and 18 years old when samples were collected in 2019 and 2021, respectively. Where possible, we sampled the same plants for the 2 years (unless the plant had died in the meantime). Some vineyards were uprooted (i.e.*,* Villette and Saillon vineyards), and some replacements were managed inconsistently (i.e.*,* Commugny). Hence, three vineyards were excluded for the second sampling year. As a result of the exclusion of certain vineyards for the reasons explained above, there were fewer plants sampled in 2021 in comparison to 2019. Each selected grapevine plant was sampled at the grafting point using a nondestructive method (43). A 0.5 cm^2^ piece of bark was removed with a surface-disinfected scalpel (80% ethyl alcohol). Next, sampling was performed using a power drill with a surface-sterilized drill bit (Ø 3.5 mm) at the spot where the bark was removed to a 1.5–2 cm depth in the trunk. The depth corresponds to the location where a discolored stripe developed during esca (51, 84) and where pathogenic activity can potentially occur (51). Chips (~60 mg) extracted by the power drill were collected in Eppendorf tubes held underneath using sterilized tweezers. Eppendorf tubes containing the coiled wood were stored at −80°C. We sampled the plants at the trunk level as the fungi associated with esca disease have never been recovered close to the leaves (30, 85). We collected apparently healthy and necrotic wood without distinction. The rationale was to not bias the recovered mycobiome by selecting wood according to appearances. This allowed us to pool apparently healthy and necrotic wood and to analyze the recovered mycobiome of asymptomatic and symptomatic plants uniformly.

### DNA extraction from wood samples

Eppendorf tubes containing wood samples and two 5 mm iron beads were placed in liquid nitrogen. The material was ruptured two times for 1 min at 30 Hz in a TissueLyser (Qiagen Inc., Germantown, MD, USA). Between and after these two steps of tissue disruption, tubes were placed in liquid nitrogen for 1 min. The tubes were placed on ice for slow thawing, and 1 mL of cetyltrimethylammonium bromide (CTAB) was added to each tube. The samples were then centrifuged for 1 min at 15,000 rpm, and the supernatant was transferred to a new tube. Fungal DNA was extracted using a Qiacube robot with the DNeasy Plant Pro Kit 69206 (Qiagen).

### Amplification of fungal ribosomal DNA

The ITS was targeted for amplification using primers ITS1F (CTTGGTCATTTAGAGGAAGTAA) and ITS4 (TCCTCCGCTTATTGATATGC) (86). We followed the PacBio procedure using barcoded universal primers for multiplexing amplicons, which includes two PCR steps (see https://www.pacb.com). The first PCR program was 30 s of denaturation at 98°C and then 30 cycles of 15 s at 98°C, 15 s at 55°C, and 1 min 30 s at 72°C, followed by a final elongation step for 7 min at 72°C. The second PCR program was 30 s of denaturation at 98°C and then 20 cycles of 15 s at 98°C, 15 s at 64°C, and 1 min 20 s at 72°C, followed by a final elongation step for 7 min at 72°C (Pacific Biosciences, 2019). The final libraries were quantified with a Qubit fluorometer (Thermo Fisher, Foster City, CA, USA), and then all samples were pooled equimolarly. Amplicons were purified and prepared for SMRT sequencing at the Functional Genomics Center in Zürich (FGCZ), Switzerland. Sequencing was performed on the PacBio Sequel II platform. Negative controls were not performed; however, previous studies have assessed the procedure and sequencing and demonstrated minimal contamination levels.

### Demultiplexing and analyses of amplicon sequence variants

Raw reads were processed with the DADA2 package in R (Callahan Github, https://github.com/benjjneb/dada2). We quality-trimmed, filtered reads, and inferred amplicon sequence variants (ASVs) with DADA2. For chimera detection, we observed a detection that was too light made by the DADA2 algorithm (isBimeraDenovo), and consequently, we used the QIIME2 uchime-denovo function. Taxonomic assignments were performed with the function *AssignTaxonomy()* of the DADA2 pipeline, which classifies sequences based on the reference training data sets and based on the UNITE general FASTA release database (2023-07-25 (87)). We calculated Chao1 indices to assess the richness and diversity of the trunk mycobiome using the *vegan* R package (88). Differences in taxonomic diversity were tested using a Wilcoxon test (*P* < 0.05).

### Analyses and characterization of taxa associated with esca

We assessed dissimilarity distances to visualize beta diversity and quantified differences in the overall ASV composition based on a principal coordinates analysis (PCoA) with Bray-Curtis distances (89). Beta diversity dissimilarities in fungal communities were assessed at the sample level, health status, and geographic region. Differences between groups in taxonomic composition were tested using three distinct methods. Lin and Peddada (90) developed ANCOM-BC and reviewed several differential abundance (DA) analysis methods. We used their review to select methods for analyzing our taxa table, including ANCOM-BC, ANCOM2, and LefSe, to test for differential abundance between asymptomatic and symptomatic plants. Linear discriminant analysis effect size (LEfSe) (59) focuses on the relationship between microbial profiles and the presence of symptoms. LEfSe is based on Kruskal-Wallis rank sum tests to identify taxa with significant differential abundance (alpha = 0.05) between groups using one-against-all comparisons. The analyses are followed by a linear discriminant analysis (LDA) to estimate the effect size of each differentially abundant feature (LDA >2). ANCOM2 based on Aitchison’s methodology uses relative abundances to infer absolute abundances (Mandal et al., 2015). Analysis of Compositions of Microbiomes with Bias Correction (ANCOM-BC) was used with an adjustment for sampling fraction by adding a sample-specific offset term in a linear regression model (90). This offset term corrects for biases, and the log-transformed linear regression framework addresses microbiome data compositionality (90). ANCOM-BC effectively controls the false discovery rate (FDR) while maintaining adequate power. Niche characteristics and traits shared by identified genera were analyzed using the FungalTraits database (54). Figure panels were generated using the R package *ggplot2* v3.3.3 (91).

## Data Availability

All PacBio sequencing data are available from the NCBI Sequence Read Archive (SRA) under BioProject number PRJNA1117061.

## SUPPLEMENTAL MATERIAL

Supplemental tables aem.00782-25-s0001.xls Tables S1 to S3.

ASM does not own the copyrights to Supplemental Material that may be linked to, or accessed through, an article. The authors have granted ASM a non-exclusive, world-wide license to publish the Supplemental Material files. Please contact the corresponding author directly for reuse.
